# Relationship between senescence in macaques and bone marrow mesenchymal stem cells and the molecular mechanism

**DOI:** 10.18632/aging.101762

**Published:** 2019-01-23

**Authors:** Xing-hua Pan, Yu-hao Chen, Yu-kun Yang, Xue-juan Zhang, Qing-keng Lin, Zi-an Li, Xue-min Cai, Rong-qing Pang, Xiang-qing Zhu, Guang-ping Ruan

**Affiliations:** ^1^ Kunming Key Laboratory of Stem Cell and Regenerative Medicine, 920th Hospital of Joint Logistics Support Force, Kunming, Yunnan Province, China; ^2^ Stem Cells and Immune Cells Biomedical Techniques Integrated Engineering Laboratory of State and Regions, Kunming, Yunnan Province, China; ^3^ Cell Therapy Technology Transfer Medical Key Laboratory of Yunnan Province, Kunming, Yunnan Province, China

**Keywords:** macaque, senescence, bone marrow mesenchymal stem cells, cytokines, transcriptome sequencing

## Abstract

The relationship between bone marrow mesenchymal stem cells (BMSCs) and aging, as well as the antiaging effects of BMSCs, was observed. An aging macaque BMSC model was established. We isolated BMSCs from young and aged macaques and used RT-PCR and Western blot to confirm the aging-related mRNAs and their expression, revealing that TERT, SIRT1 and SIRT6 expression was decreased in the aged BMSCs. The morphology, immunophenotype, differentiation potential, proliferation potential, and antiaging effects of aged and young BMSCs on 293T cells were compared. The expression of aging-related genes and the difference between the secreted cytokines in natural aging and induced aging BMSCs were observed. The transcriptome of peripheral blood mononuclear cells from macaques was analyzed by high-throughput sequencing. Finally, the transcriptional characteristics and regulatory mechanisms of gene transcription in aging macaques were investigated.

## INTRODUCTION

Over the next 50 years, the aging population in developing countries is expected to grow four-fold. Since the end of the 20^th^ century, China has gradually become an aging society. The problems brought on by aging have become more prominent and pose a serious challenge to the development of the entire society. Therefore, it is urgent to deal with the diseases caused by aging and the aging body. Aging is a natural law that cannot be avoided by individuals. The body is chronically affected by various environmental factors inside and outside the body, causing the structure and function of various tissues and organs to gradually degenerate, eventually developing chronic tissue syndromes or organ dysfunction [1]. However, the mechanism of aging has not been thoroughly elucidated to date. In recent years, the rapid development of cell biotherapy has led to new opportunities to elucidate the diseases related to the elderly. The role of cell biotherapy in this regard has attracted the attention of researchers and achieved important progress.

Adult stem cells play an essential role in tissue engineering research and are widely used to treat degenerative diseases. Stem cells can self-renew and differentiate and are seed cells that maintain the structure and function of tissues and organs. The whole process of human growth, development, aging and death involves the proliferation and differentiation of stem cells. The physiological replacement of tissues and the repair of pathological damage are also dependent on the mobilization, proliferation and differentiation of stem cells [2]. The aging of stem cells is an important cause of the decline in tissue and organ function and is also an important factor affecting the efficacy of stem cell therapy in aging patients [3]. The aging of the human body is mainly due to the decrease in the number of stem cells and their activity, resulting in fewer young cells than aged cells, a slow metabolism and, subsequently, degenerative diseases [4]. Signer et al. showed that aging is a gradual, cumulative process. The normal operation and environmental exposure of the body may cause damage. Therefore, the body needs to be repaired continuously. In embryonic development and human growth, stem cells have robust proliferation and differentiation ability; therefore, repairs are easy at these stages. However, in aging tissues and organs, the activity of stem cells is markedly decreased. With increasing age, the number and activity of stem cells in the body are decreased, the appearance of cells in some tissues and organs is lost, and a novel supply of cells is not obtained. This change will affect the function of the organism, eventually leading to functional failure of important organs and even death [5].

Mesenchymal stem cells (MSCs) are heterogeneous adult stem cells that were originally isolated from the bone marrow and later extracted from the connective tissue of almost all organs. Professor P. Bianco and colleagues [6–11] argued that the trilineage MSCs in bone marrow are skeletal progenitors that differ from MSC-like cells in other tissues. It is clear that non-BMSCs may be more accurately referred to as MSC-like cells. MSCs have good self-renewal ability and the ability to differentiate into a variety of mesodermal cells, such as adipocytes, osteoblasts and chondrocytes, which serve as functional criteria for identifying MSCs [12].

MSCs exhibit a variety of biological functions, including proper immune regulation and secretion of growth factors, cytokines and angiogenic mediators. These cells have immunosuppressive functions. Therefore, we believe that MSCs may be an effective therapeutic for immune mediator-related diseases [13, 14]. By expressing a variety of chemokines and chemokine receptors, MSCs migrate towards inflammatory chemokines and cytokines, reach the area where inflammation occurs, and promote tissue regeneration and repair [15, 16]. A phase I multicenter clinical study and phase II clinical trial studies have shown that bone marrow mesenchymal stem cells (BMSCs) can be safely applied to the treatment of hormone-resistant graft-versus-host disease (GVHD) by intravenous injection without acute poisoning [17].

However, with age, the risk of bone marrow failure increases and tolerance to cytotoxic damage is reduced. As the body ages, MSCs will show signs of aging, which limits the use of autologous MSCs in transplant treatment for elderly patients [18]. Understanding the development and mechanism of aging BMSCs is vital in basic research and can be applied to clinical practice.

Macaques diverged from humans approximately 25 million years ago, and the similarity of their genome to that of humans is approximately 93% [19]. Macaques are widely used in basic and applied biomedical research, which has greatly promoted the development of multidisciplinary research.

Understanding the molecular mechanisms of aging BMSCs will help to identify more effective diagnostic and therapeutic strategies. Through the analysis of biological functions and signaling pathways, we can further understand the changes in aging at the molecular level to explore the potential diagnostic, prognostic and biological markers of drug targets, which is of great significance for preclinical research on aging.

This study attempts to compare the differences in miRNA and mRNA gene expression levels between juvenile and aged macaque mononuclear cells by second-generation sequencing technology. The differences in peripheral blood cells and bone marrow cytokines were observed. The bone marrow cells of juvenile and aging macaques were extracted. BMSCs were isolated for culture and biocharacteristics analysis. The aging cell model was established using the strong oxidant t-BHP. The telomere length and telomerase expression in target cells were affected by cell coculture, and the aging of macaque BMSCs and mononuclear cells was explored by modern biological techniques. The changes in biological characteristics provide a valuable theoretical basis for research on and the application of macaques and their BMSCs.

## RESULTS

### Morphological characteristics of BMSCs

**Figure 1:**
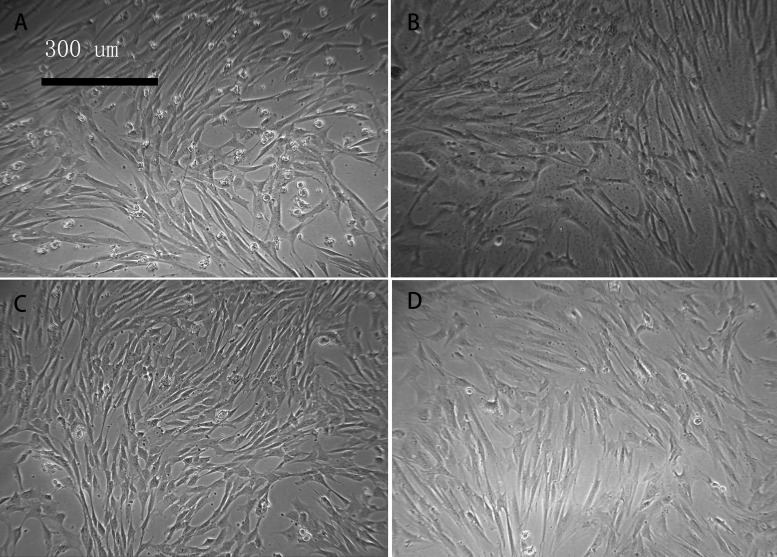
**Cell morphology of young and aged macaque BMSCs (×100).** (**A**, **B**) show the primary BMSCs of young and aged macaques, respectively; (**C**, **D**) show the P3 BMSCs of young and aged macaques, respectively. With prolonged in vitro culture, some cells in the aging group became broad, and the number of polygonal and irregularly shaped cells began to increase.

With prolonged in vitro culture, some cells in the aging group became broad, and the number of polygonal and irregularly shaped cells began to increase. The nuclear-to-cytoplasmic ratio of the cells decreased. In addition, there were small particles or small vacuoles observed in the cytoplasm. The shape of the cells in the young group was relatively uniform, with a strong refractive index and optimal growth (Figure 1).

### Immunophenotype of macaque BMSCs

Flow cytometry was performed on P3 BMSCs, and the cell surface antigens CD29, CD105, CD73, CD184, CD90, CD45 and HLA-DR were detected. There were several differences in the surface antigens of the BMSCs in the young and aged groups, but these differences were not statistically significant (Table 1). Only CD90 showed a significant difference (P < 0.05). The percentage of CD90-positive cells (99.43 ± 0.45%) of young macaques was significantly higher than that of aged macaques (24.37 ± 17.35%).

**Table 1:** Flow cytometry analysis of surface antigens in young and aged macaque BMSCs ($\bar{X}\pm \text{s}$)

Surface antigens	Groups	Positive cell rate(%)	P-value
CD29	Young cell group	100.00 ± 0.00	0.423
	Aged cell group	99.97 ± 0.06	
CD105	Young cell group	99.83 ± 0.06	0.057
	Aged cell group	99.97 ± 0.06	
CD73	Young cell group	99.97 ± 0.06	0.329
	Aged cell group	98.60 ± 1.85	
CD184	Young cell group	96.83 ± 2.85	0.409
	Aged cell group	88.2 ± 15.96	
CD90	Young cell group	99.43 ± 0.45	0.017
	Aged cell group*	24.37 ± 17.35	
CD45	Young cell group	0.17 ± 0.12	0.097
	Aged cell group	3.47 ± 2.65	
HLA-DR	Young cell group	0.33 ± 0.23	0.066
	Aged cell group	4.53 ± 2.89	

**Note:** *P < 0.05 compared with the young cell group (n=3).

### Ability to induce differentiation of macaque BMSCs

**Figure 2:**
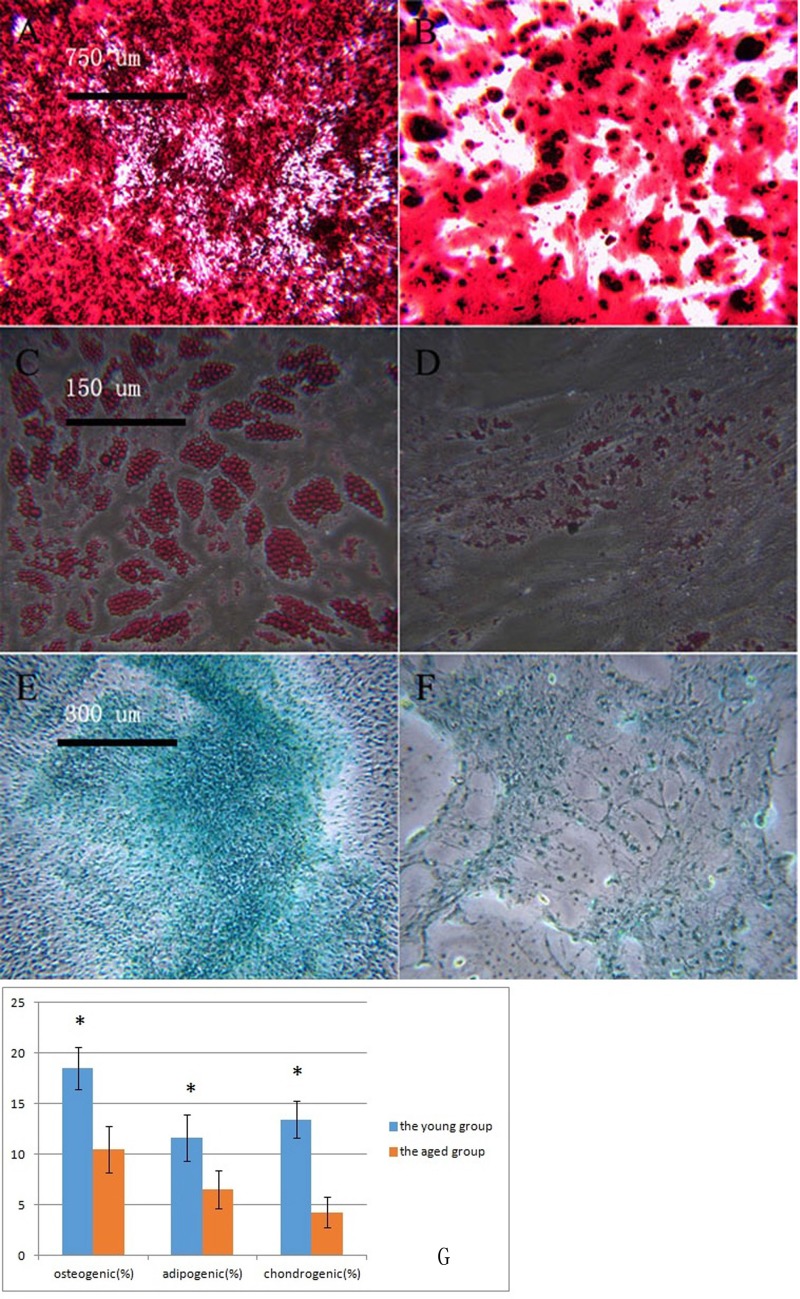
**Macaque BMSCs differentiated into multiple cells.** (**A**, **B**) show the osteogenic induction of young and aged macaque BMSCs, respectively (×40); (**C**, **D**) show the adipogenic induction of young and aged macaque BMSCs, respectively (×200); (**E**, **F**) show the cartilage induction of young and aged macaque BMSCs, respectively. (×100); (**G**) We used ImageJ 1.37c software to quantitate the osteogenic, adipogenic and chondrogenic results. In the aged group, less osteogenic, adipogenic and chondrogenic markers were produced than in the young group (^*^p<0.05, n=3).

P3 BMSCs in the young and aged groups underwent osteogenic induction. After staining with alizarin red, the dark-red calcium nodules were quantified (Figure 2A, 2B). The number of calcium nodules in the aged group was fewer than that in the young group. In the young group and the aged group, the macaque BMSCs underwent adipogenesis. After adding the oil red O stain, the dye was dissolved in the intracellular lipids, and the lipid droplets in the cells were red (Figure 2C, 2D). Fewer lipid droplets were produced in the aged group than in the young group. In the young group and the aged group, the macaque BMSCs underwent cartilage induction, and alcian blue staining showed the proteoglycans synthesized by the blue chondrocytes (Figure 2E, 2F). The aged group had less proteoglycans than the young group. We then used ImageJ 1.37c software to quantitate the osteogenic, adipogenic and chondrogenic results, which are shown in Figure 2G. Overall, in the aged group, the osteogenic, adipogenic and chondrogenic markers produced were less than those in the young group (^*^p<0.05, n=3).

### Proliferative ability of macaque P3 BMSCs

**Figure 3:**
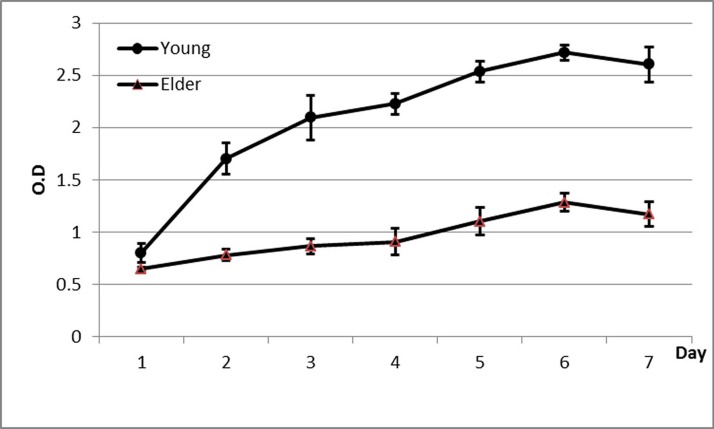
**Proliferation curve of BMSCs from aged and young macaques as determined by the CCK-8 method.** It was observed that compared with that in the young group, the expansion rate of P3 BMSCs in the aged group was significantly lower, and the proliferative ability was significantly reduced.

It was observed that compared with that in the young group, the expansion rate of P3 BMSCs in the aged group was significantly lower, and the proliferative ability was significantly reduced (Figure 3, Table 2, P < 0.05).

**Table 2:** Proliferation of BMSCs from aged and young macaques by the CCK-8 assay

	n	Aged group	Young group	P-value
Day 1	3	0.65 ± 0.02*	0.80 ± 0.09	0.004
Day 2	3	0.78 ± 0.06*	1.70 ± 0.15	<0.001
Day 3	3	0.87 ± 0.07*	2.10 ± 0.22	<0.001
Day 4	3	0.91 ± 0.12*	2.23 ± 0.10	<0.001
Day 5	3	1.10 ± 0.13*	2.53 ± 0.10	<0.001
Day 6	3	1.28 ± 0.08*	2.72 ± 0.07	<0.001
Day 7	3	1.17 ± 0.12*	2.60 ± 0.17	<0.001

Note: *Compared with the young group, P < 0.01.

### Detection of senescence-related SA-β-gal in macaque BMSCs

**Figure 4:**
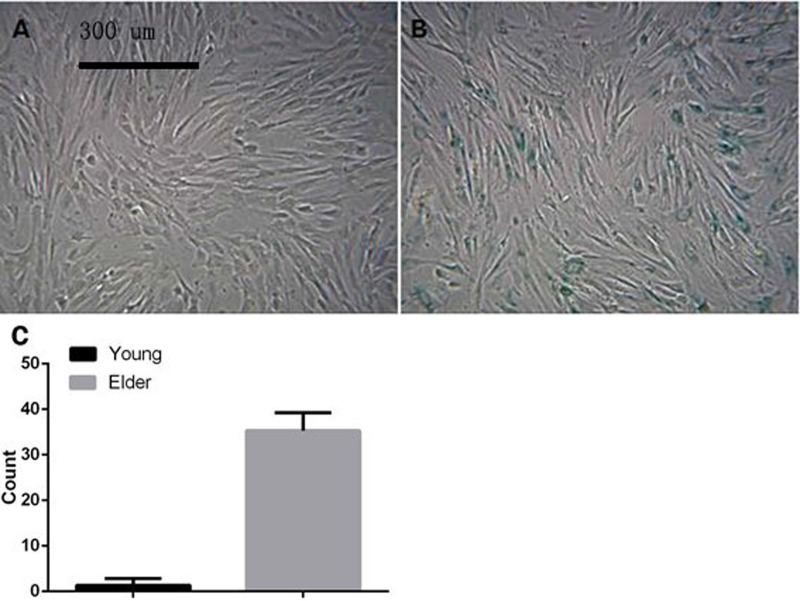
**Macaque P3 BMSCs stained with senescence-associated SA-β-gal (A) and (B) show young macaque and aged macaque P3 BMSCs, respectively (×100).** The results showed that the percentage of positive BMSC staining in the young group was 1.33 ± 1.51%, which was significantly lower than that in the aged group (35.33 ± 3.88%, p<0.01), suggesting that the BMSCs in aged macaques exhibit cell senescence earlier than those in young macaques.

P3 BMSCs from young and aged macaques were stained with SA-β-gal. The results showed that the percentage of positive BMSC staining in the young group was 1.33 ± 1.51%, which was significantly lower than that in the aged group (35.33 ± 3.88%, p<0.01, Figure 4), suggesting that the BMSCs in aged macaques exhibit cell senescence earlier than those in young macaques.

### Antiaging role of macaque BMSCs in cocultures

### *Results of the induction of senescence in 293T cells*

**Figure 5:**
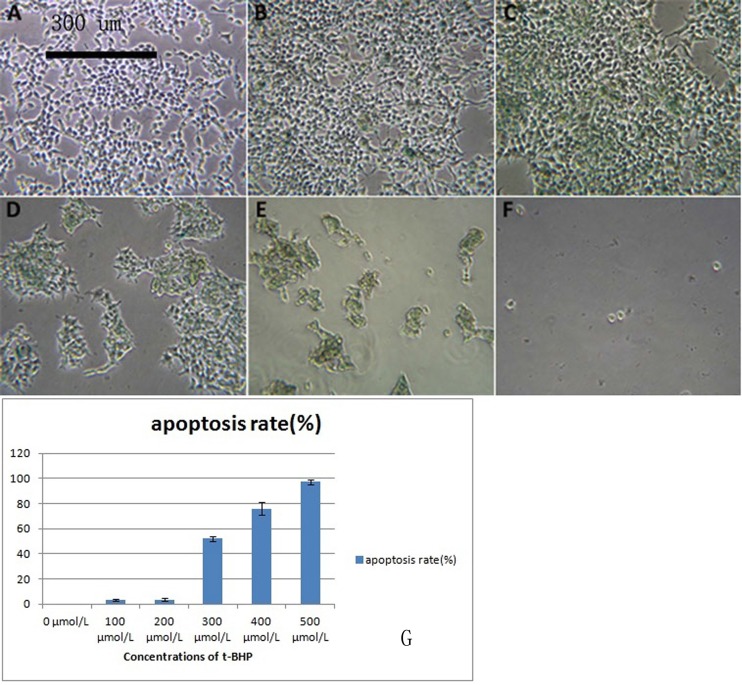
**293T cells treated with different concentrations of t-BHP and stained with SA-β-gal (×100).** BMSCs were treated with t-BHP at concentrations of 0 μmol/L (**A**), 100 μmol/L (**B**), 200 μmol/L (**C**), 300 μmol/L (**D**), 400 μmol/L (**E**), and 500 μmol/L (**F**). We have quantified the apoptosis rate in (**G**). Induction with 200 μmol/L t-BHP for 6 h was performed to establish the aging macaque BMSC model.

After induction by different concentrations of t-BHP, cells showed different apoptosis rates, and some cells showed senescence after induction (Figure 5A–5G). Induction with 200 μmol/L t-BHP for 6 h was performed to establish an aging model with macaque BMSCs.

Flow cytometry analysis of 293T cells before and after the induction of senescence showed that the detection rates of the surface antigens CD29, CD73, CD105 and HLA-ABC in senescent 293T cells were significantly decreased (Table 3, P < 0.05).

**Table 3:** Flow cytometry analysis of surface antigens in 293T cells before and after senescence induction ($\bar{X}\pm \text{s}$, n = 3)

Surface antigens	Groups	Positive cell rate(%)	**P-value**
CD14	Normal group	78.8 ± 0.8	0.089
	Induction group	76.3 ± 1.7	
CD29	Normal group	88.3 ± 1.1	0.009
	Induction group^**^	84.5 ± 0.9	
CD90	Normal group	75.5 ± 1.0	0.078
	Induction group	72.7 ± 1.8	
CD73	Normal group	4.55 ± 0.53	0.010
	Induction group^*^	2.77 ± 0.42	
CD105	Normal group	83.2 ± 2.1	0.008
	Induction group^**^	68.4 ± 4.8	
HLA-ABC	Normal group	1.12 ± 0.04	<0.001
	Induction group^**^	0.19 ± 0.02	

**Note:** ^*^Compared with the normal group, P < 0.05, and ^**^ compared with the normal group, P < 0.01 (n=3).

### *Telomerase and TCAB1 mRNA expression levels in 293T cells*

**Figure 6:**
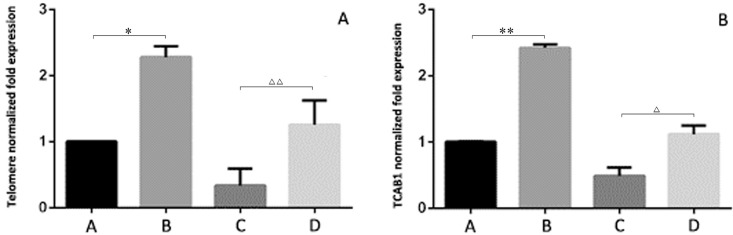
**Relative expression levels of telomeres and TCAB1 in 293T cells by RT-PCR.** (**A**) shows the senescence-induced 293T cells cocultured with BMSCs. (**B**) shows the control 293T cells cocultured with BMSCs. (**C**) shows the senescence-induced 293T cells. (**D**) shows the control 293T cells. After the 293T cells were cocultured with BMSCs for 5 d, the mRNA levels of telomeres in the control group and the induced senescence group were significantly higher than those measured before coculture (P < 0.05, Figure 6A), and the mRNA levels of TCAB1 were also significantly increased (P < 0.05, Figure 6B).

After the 293T cells were cocultured with BMSCs for 5 d, the mRNA levels of telomeres in the control group and the induced senescence group were significantly higher than those measured before coculture (P < 0.05, Figure 6A), and the mRNA levels of TCAB1 were also significantly increased (P < 0.05, Figure 6B).

### *TCAB1 protein expression levels in 293T cells*

**Figure 7:**
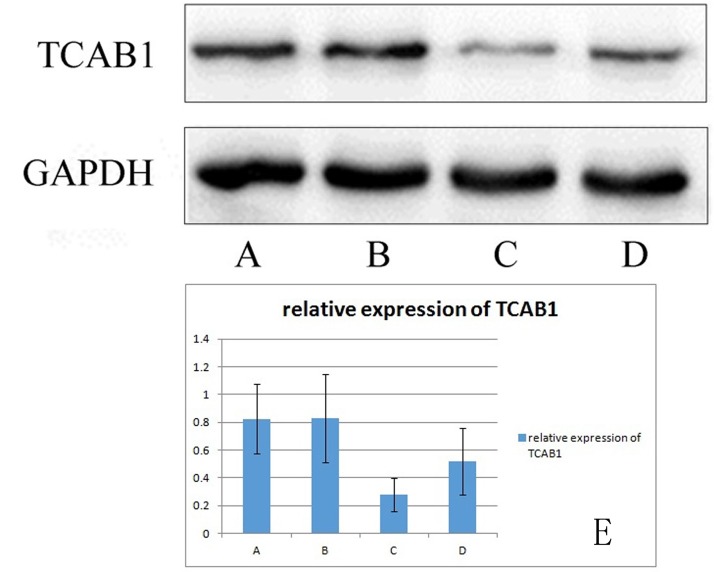
**Relative expression of TCAB1 in 293T cells.** (**A**) shows TCAB1 expression in senescence-induced 293T cells cocultured with BMSCs. (**B**) shows TCAB1 expression in control 293T cells cocultured with BMSCs. (**C**) shows TCAB1 expression in senescence-induced 293T cells, and (**D**) shows TCAB1 expression in control 293T cells. (**E**) shows the Western blots with the quantitated results. TCAB1 expression was weakly positive before induction and positive after induction.

TCAB1 is expressed in 293T cells. After coculture with BMSCs, the expression level of TCAB1 was significantly higher than that before coculture. The TCAB1 expression level was weakly positive before induction and positive after induction (Figure 7).

### Results of induced aging in macaque BMSCs

**Figure 8:**
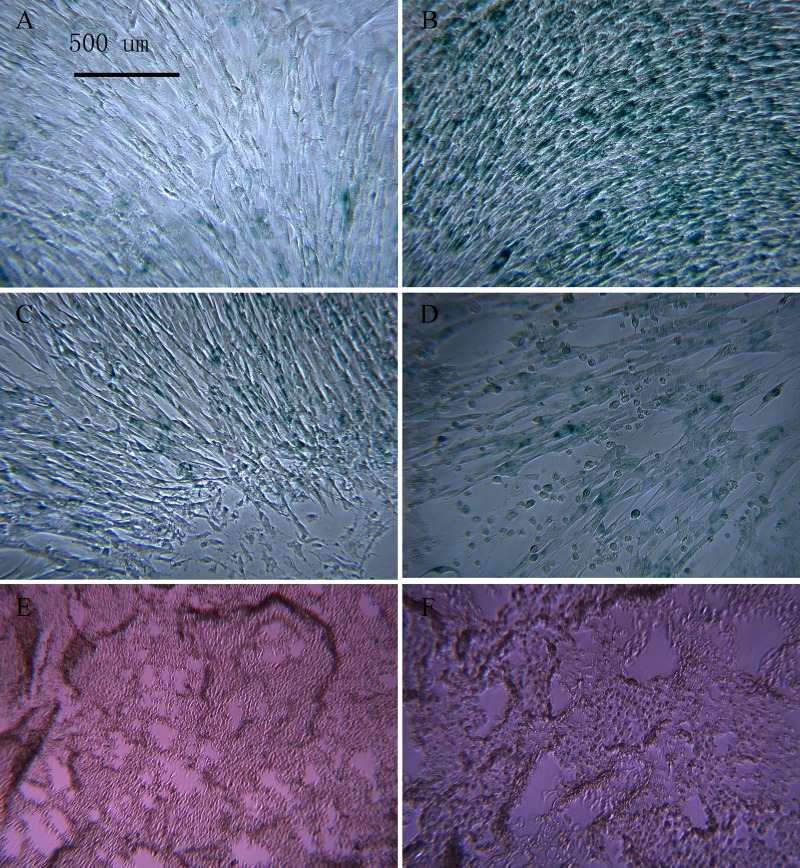
**BMSCs treated with different concentrations of t-BHP and stained with SA-β-gal (×100).** The concentration of t-BHP used to treat BMSCs is 100 μmol/L (**A**), 200 μmol/L (**B**), 300 μmol/L (**C**), 400 μmol/L (**D**), and 500 μmol/L (**E**), and 600 μmol/L (**F**). The aging macaque BMSC model was established by inducing cells with 200 μmol/L t-BHP for 6 h.

After induction with different concentrations of t-BHP for 6 h, SA-β-gal staining showed that some cells were senescent at a concentration of 200 μmol/L; the rate of positive staining was 34.0 ± 4.0%, and the number of SA-β-gal-positive cells was significantly higher than that in the other groups (P < 0.05). No cell death was observed. Therefore, the aging macaque BMSC model was established by exposure to 200 μmol/L t-BHP for 6 h (Figure 8).

### Detection of cytokine content in the culture supernatant of macaque BMSCs

**Figure 9:**
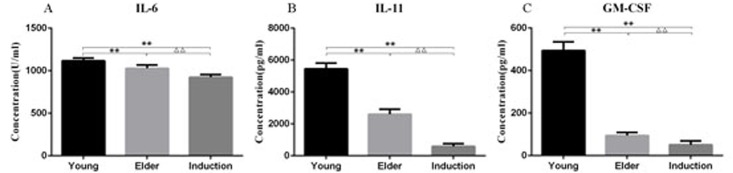
**Cytokine levels in the culture supernatant of the young, aged and induced aged macaque BMSCs.** IL-6, IL-11 and GM-CSF levels were significantly decreased in the elderly group and the induced aging group compared with the juvenile group (P < 0.01). IL-6, IL-11 and GM-CSF levels were also significantly lower in the induced aging group than in the elderly group (P < 0.01).

The BMSC culture supernatants from the juvenile cell group, old cell group and induced aging group were analyzed by ELISA. IL-6, IL-11 and GM-CSF levels were significantly decreased in the elderly group and the induced aging group compared with the juvenile group (P < 0.01). IL-6, IL-11 and GM-CSF levels were also significantly lower in the induced aging group than in the elderly group (P < 0.01, Figure 9).

### RT-PCR detection of senescence-associated mRNA expression levels in BMSCs

**Figure 10:**
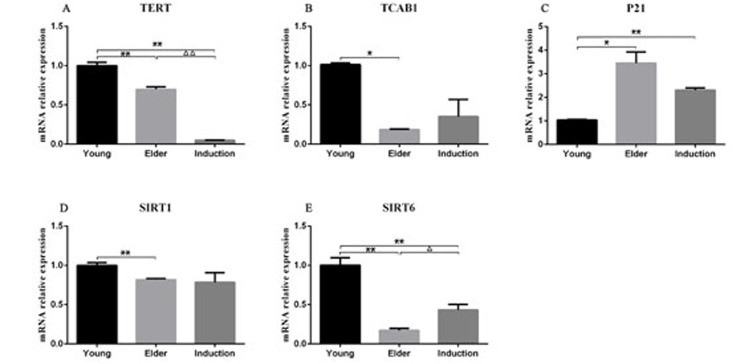
**RT-PCR detection of the relative expression of TERT, TCAB1, P21, SIRT1 and SIRT6 in BMSCs.** The results showed that TERT, SIRT1 and SIRT6 expression levels were significantly lower in the aged cell group than in the juvenile cell group (P < 0.01). P21 expression was significantly increased (P < 0.01), and TCAB1 expression was significantly decreased (P < 0.05). Compared with those in the juvenile cell group, TERT and SIRT6 levels were significantly decreased (P < 0.01) and P21 levels were significantly increased (P < 0.01) in the induced senescence group. TERT expression was significantly decreased in the induced aging group compared with the aged cell group (P < 0.01), and SIRT6 expression was significantly decreased (P < 0.05).

RT-PCR was used to detect the expression levels of senescence-associated mRNA in the juvenile cell group, old cell group and induced senescence group (Table 4). The results showed that TERT, SIRT1 and SIRT6 levels were significantly lower in the aged cell group than in the juvenile cell group (P < 0.01, Figure 10A, 10D, 10E). The level of P21 was significantly increased (P < 0.01, Figure 10C), and the level of TCAB1 was significantly decreased (P < 0.05, Figure 10B). Compared with those in the juvenile cell group, TERT and SIRT6 levels were significantly decreased (P < 0.01, Figure 10A) and P21 levels were significantly increased (P < 0.01, Figure 10C) in the induced senescence group. The level of TERT was significantly decreased in the induced aging group compared with the aged cell group (P < 0.01, Figure 10A), and that of SIRT6 was significantly decreased (P < 0.05, Figure 10E).

**Table 4:** Relative expression levels of senescence-associated mRNA in each group of BMSCs ($\bar{X}\pm \text{s}$)

	n	TERT	TCAB1	P21	SIRT1	SIRT6
Young cell group	3	1.00 ± 0.04	1.01 ± 0.02	1.03 ± 0.03	1.00 ± 0.04	1.00 ± 0.09
Aged cell group	3	0.70 ± 0.03^**^	0.18 ± 0.01^*^	3.45 ± 0.48^**^	0.82 ± 0.02^**^	0.17 ± 0.02^**^
Induced aged group	3	0.05 ± 0.00^**△△^	0.35 ± 0.22	2.31 ± 0.09^**^	0.78 ± 0.12	0.43 ± 0.07^**△△^

**Note:** ^*^Compared with the young cell group, P < 0.05, ^**^compared with the young cell group, P < 0.01, △compared with the aged cell group, P < 0.05, and △△compared with the aged cell group, P < 0.01.

### Western blot analysis of aging BMSC-related protein expression

**Figure 11:**
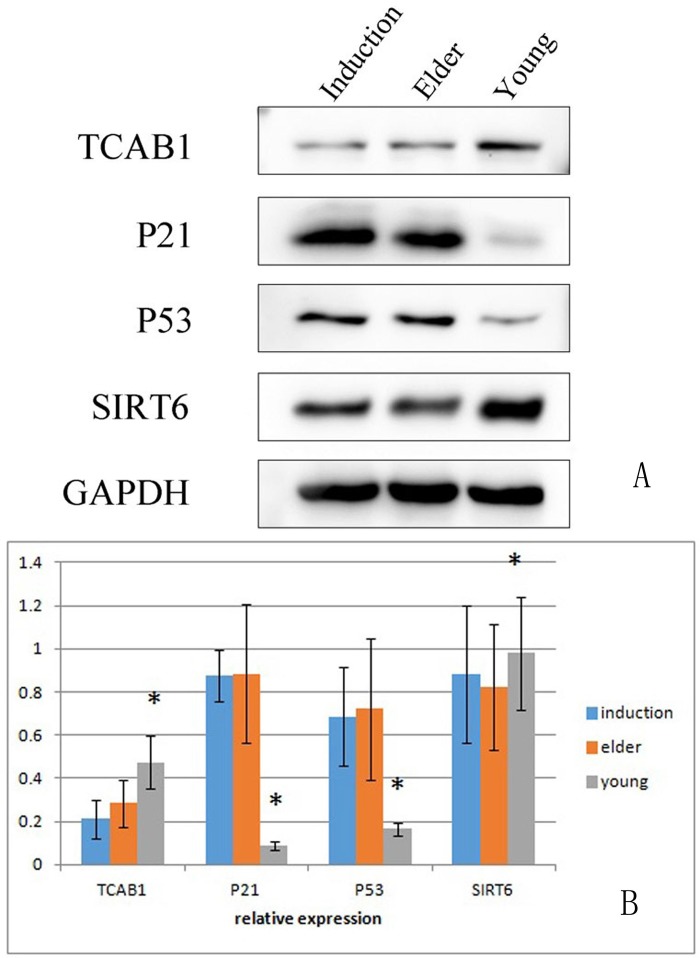
**Expression of aging-associated proteins in the young cell group, aged cell group and induced aged BMSC group.** The levels of TCAB1 and SIRT6 were relatively low in the aged and induced aging groups compared with the young group. p21 and p53 expression levels were relatively high in the aged and induced aging groups. ^*^p<0.05 compared to the other two groups.

Western blotting was used to detect the expression of senescence-associated proteins in juvenile cells, aged cells, and senescence-induced BMSCs. The levels of TCAB1 and SIRT6 were relatively low in the aged and induced aging groups compared with those in the young group. p21 and p53 levels were relatively high (Figure 11) in the aged and induced aging groups. ^*^p<0.05 compared to the other two groups.

### Angiogenic factors in macaques

There were significant differences in angiogenesis-related factors between the juvenile group and the old group of macaques (P < 0.05). The levels of HB-EGF and PIGF in the juvenile group were lower than those in the old group, while the levels of VEGF in the juvenile group were higher than those in the old group (Table 5).

**Table 5-1:** Quantification of angiogenesis-related factors in juvenile and aged macaque ($\bar{X}\pm \text{s}$, pg/ml)

	n	Angiogenin	ANG-2	EGF	bFGF	HB-EGF
Young group	3	0.29 ± 0.50	112.22 ± 95.59	0.02 ± 0.02	17.22 ± 29.83	4.74 ± 0.88
Aged group	3	27.70 ± 38.30	138.10 ± 55.38	0.28 ± 0.24	191.90 ± 177.63	8.99 ± 0.86^*^

**Table 5-2:** Quantification of angiogenesis-related factors in juvenile and aged macaques ($\bar{X}\pm \text{s}$, pg/ml)

	n	HGF	Leptin	PDGF-BB	PIGF	VEGF
Young group	3	17.62 ± 25.21	60.46 ± 41.09	7.50 ± 7.02	0.18 ± 0.13	4.03 ± 1.20
Aged group	3	0.70 ± 1.18	337.42 ± 529.09	11.46 ± 13.29	3.24 ± 0.72^*^	0.00 ± 0.00^*^

**Note:** ^*^Compared with the juvenile group, P < 0.05. The levels of HB-EGF and PIGF in the juvenile group were lower than those in the old group, while the levels of VEGF in the juvenile group were higher than those in the old group.

### Peripheral blood cell analysis

The number of white blood cells and lymphocytes isolated from the peripheral blood of old macaques was significantly increased (P < 0.05) and higher than the normal reference values. The other indicators showed no significant changes (Table 6). The results suggest that elderly macaques may be in a state of chronic inflammation.

**Table 6-1:** Analysis of peripheral blood cells from young and aged macaques ($\bar{X}\pm \text{s}$, × 10^9^/L)

	n	Leukocyte	Neutrophil granulocyte	Lymphocyte	Monocyte
Young group	3	8.1 ± 1.5	3.5 ± 0.7	4.3 ± 1.5	0.17 ± 0.05
Aged group	3	12.4 ± 1.6^*^	3.9 ± 1.3	8.2 ± 1.5^*^	0.24 ± 0.05

**Note:** ^*^Compared with the juvenile group, P < 0.05. The results suggest that elderly macaques may be in a state of chronic inflammation.

**Table 6-2:** Analysis of peripheral blood cells from young and aged macaques ($\bar{X}\pm \text{s}$)

	n	Red blood cell	Blood platelets
Young group	3	(6.2 ± 0.9) × 10^12^/L	(359.3 ± 12.5) × 10^9^/L
Aged group	3	(5.7 ± 0.1) × 10^12^/L	(353.0 ± 60.5) × 10^9^/L

### Transcriptome sequencing results of macaque peripheral blood mononuclear cells

**Figure 12:**
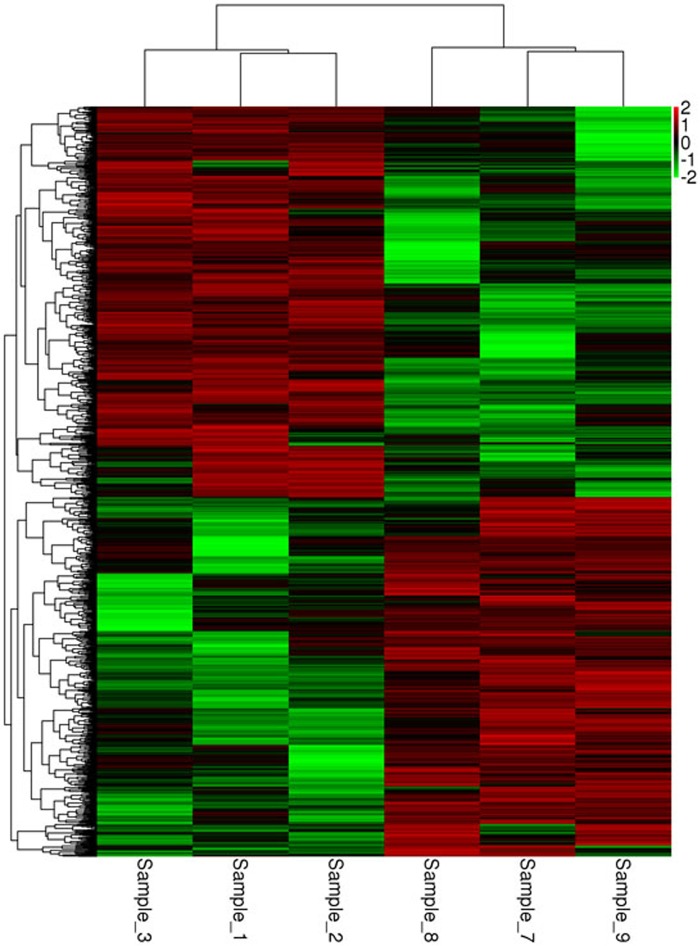
**Heat map of the mRNA clustering analysis of macaque mononuclear cells from juvenile and old groups.** Samples 1, 2, and 3 correspond to the elderly group, and samples_7, 8, and 9 correspond to the young group. According to the analysis, a total of 5,711 differentially expressed GO terms were identified, of which 2,636 were downregulated and 3,075 were upregulated.

**Figure 13:**
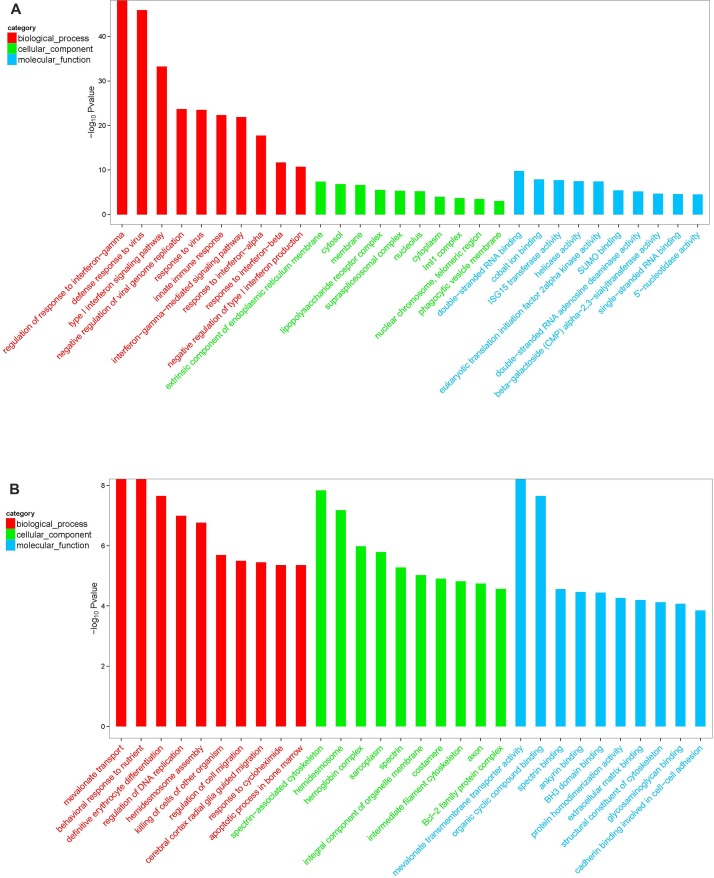
**GO enrichment analysis of differentially expressed mRNA in macaque mononuclear cells from the juvenile and old age groups.** (**A**) Downregulated GO terms. (**B**) Upregulated GO terms. Red indicates the biological process-related GO term; green indicates the cell component-related GO term; and blue indicates the molecular function-related GO term. The top 30 bar graphs of the GO enrichment analysis are shown in Figure 13A and 13B. The top 30 downregulated and upregulated GO terms are listed in Table 7 according to their P-value and sorted from small to large.

Differentially expressed mRNA was analyzed by sequencing peripheral blood mononuclear cells from the juvenile and the aged groups. The clustered genes, which may have similar biological functions, are displayed in a heat map (Figure 12). According to the analysis, a total of 5,711 differentially expressed GO terms were detected, of which 2,636 were downregulated and 3,075 were upregulated. The GO terms of the three groups with corresponding gene numbers greater than two were screened. Each of the 10 items was ranked by the associated -log10^ (*P*-value)^ value. The top 30 bar graphs of the GO enrichment analysis are shown in Figure 13A and 13B. The top 30 downregulated and upregulated GO terms are listed in Table 7 according to their P-value and sorted from small to large. The gene functions involved in the downregulated GO terms mainly included regulation of the IFN-γ response, defense against viruses, the type I IFN signaling pathway, replication of the negative regulatory viral genome, response to viruses, innate immune response, the IFN-γ signaling pathway, the response of IFN-α, the response of IFN-β, and the negative regulation of type I IFN product. The gene functions involved in the upregulated GO terms mainly included the transport of mevalonate and the behavioral response to nutrition, spectrin-related cytoskeleton, organic cyclic compound binding, directed red blood cell differentiation, hemidesmosome, regulation of DNA replication, hemidesmosome assembly, other microorganisms that kill cells, and regulation of cell migration.

**Table 7:** GO analysis of aging-related differentially expressed genes

Expression	Term	Gene function	Gene count	Enrichment score	P -value
Down	GO:0060330	Regulation of response to interferon-gamma	3	89.23	0
Down	GO:0051607	Defense response to virus	54	14.34	1.3E-46
Down	GO:0060337	Type I interferon signaling pathway	33	19.25	6.2E-34
Down	GO:0045071	Negative regulation of viral genome replication	22	20.88	2.1E-24
Down	GO:0009615	Response to virus	30	11.74	3.4E-24
Down	GO:0045087	Innate immune response	50	5.49	4.8E-23
Down	GO:0060333	Interferon-gamma-mediated signaling pathway	27	12.29	1.4E-22
Down	GO:0035455	Response to interferon-alpha	13	31.35	2.0E-18
Down	GO:0035456	Response to interferon-beta	9	24.34	2.1E-12
Down	GO:0032480	Negative regulation of type I interferon production	11	14.02	2.1E-11
Down	GO:0003725	Double-stranded RNA binding	15	7.65	1.8E-10
Down	GO:0050897	Cobalt ion binding	5	27.88	1.4E-08
Down	GO:0042296	ISG15 transferase activity	4	39.66	2.1E-08
Down	GO:0004386	Helicase activity	14	5.68	3.6E-08
Down	GO:0004694	Eukaryotic translation initiation factor 2alpha kinase activity	4	35.69	4.2E-08
Down	GO:0042406	Extrinsic component of endoplasmic reticulum membrane	5	23.48	4.6E-08
Down	GO:0005829	Cytosol	147	1.48	1.6E-07
Down	GO:0016020	Membrane	96	1.66	2.5E-07
Down	GO:0046696	Lipopolysaccharide receptor complex	3	26.77	3.1E-06
Down	GO:0032183	SUMO binding	5	11.74	3.9E-06
Down	GO:0044530	Supraspliceosomal complex	3	24.33	4.8E-06
Down	GO:0005730	Nucleolus	45	1.98	6.1 E-06
Down	GO:0003726	Double-stranded RNA adenosine deaminase activity	3	22.31	7.2E-06
Down	GO:0003836	Beta-galactoside (CMP) alpha-2,3-sialyltransferase activity	4	11.51	2.3E-05
Down	GO:0003727	Single-stranded RNA binding	6	6.86	2.8E-05
Down	GO:0008253	5'-Nucleotidase activity	4	10.82	3.2E-05
Down	GO:0005737	Cytoplasm	207	1.24	0.0001
Down	GO:1990130	Iml1 complex	3	9.91	0.0002
Down	GO:0000784	Nuclear chromosome, telomeric region	9	3.40	0.0004
Down	GO:0030670	Phagocytic vesicle membrane	6	3.88	0.0010
Up	GO:0015728	Mevalonate transport	3	81.66	0
Up	GO:0051780	Behavioral response to nutrient	3	81.66	0
Up	GO:0015130	Mevalonate transmembrane transporter activity	3	81.66	0
Up	GO:0014731	Spectrin-associated cytoskeleton	10	9.07	1.5E-08
Up	GO:0097159	Organic cyclic compound binding	3	61.24	2.2E-08
Up	GO:0060318	Definitive erythrocyte differentiation	3	61.24	2.2E-08
Up	GO:0030056	Hemidesmosome	8	10.54	6.7E-08
Up	GO:0006275	Regulation of DNA replication	8	10.05	1.0E-07
Up	GO:0031581	Hemidesmosome assembly	8	9.47	1.7E-07
Up	GO:0031640	Killing of cells of other organism	4	18.15	2.0E-06
Up	GO:0030334	Regulation of cell migration	12	4.54	3.2E-06
Up	GO:0021801	Cerebral cortex radial glia guided migration	4	16.33	3.6E-06
Up	GO:0005833	Hemoglobin complex	4	20.41	1.1E-06
Up	GO:0016528	Sarcoplasm	6	10.42	1.6E-06
Up	GO:0046898	Response to cycloheximide	3	24.50	4.4E-06
Up	GO:0071839	Apoptotic process in bone marrow	3	24.50	4.4E-06
Up	GO:0008091	Spectrin	7	7.33	5.3E-06
Up	GO:0031301	Integral component of organelle membrane	4	13.61	9.5E-06
Up	GO:0043034	Costamere	9	5.10	1.2E-05
Up	GO:0045111	Intermediate filament cytoskeleton	11	4.20	1.5E-05
Up	GO:0030424	Axon	24	2.49	1.8E-05
Up	GO:0097136	Bcl-2 family protein complex	3	16.33	2.7E-05
Up	GO:0030507	Spectrin binding	9	4.65	2.8E-05
Up	GO:0030506	Ankyrin binding	8	5.03	3.5E-05
Up	GO:0051434	BH3 domain binding	3	15.31	3.6E-05
Up	GO:0042803	Protein homodimerization activity	37	1.94	5.5E-05
Up	GO:0050840	Extracellular matrix binding	5	7.29	6.4E-05
Up	GO:0005200	Structural constituent of cytoskeleton	14	3.04	7.5E-05
Up	GO:0005539	Glycosaminoglycan binding	4	8.83	8.5E-05
Up	GO:0098641	Cadherin binding involved in cell-cell adhesion	24	2.19	0.0001

**Figure 14:**
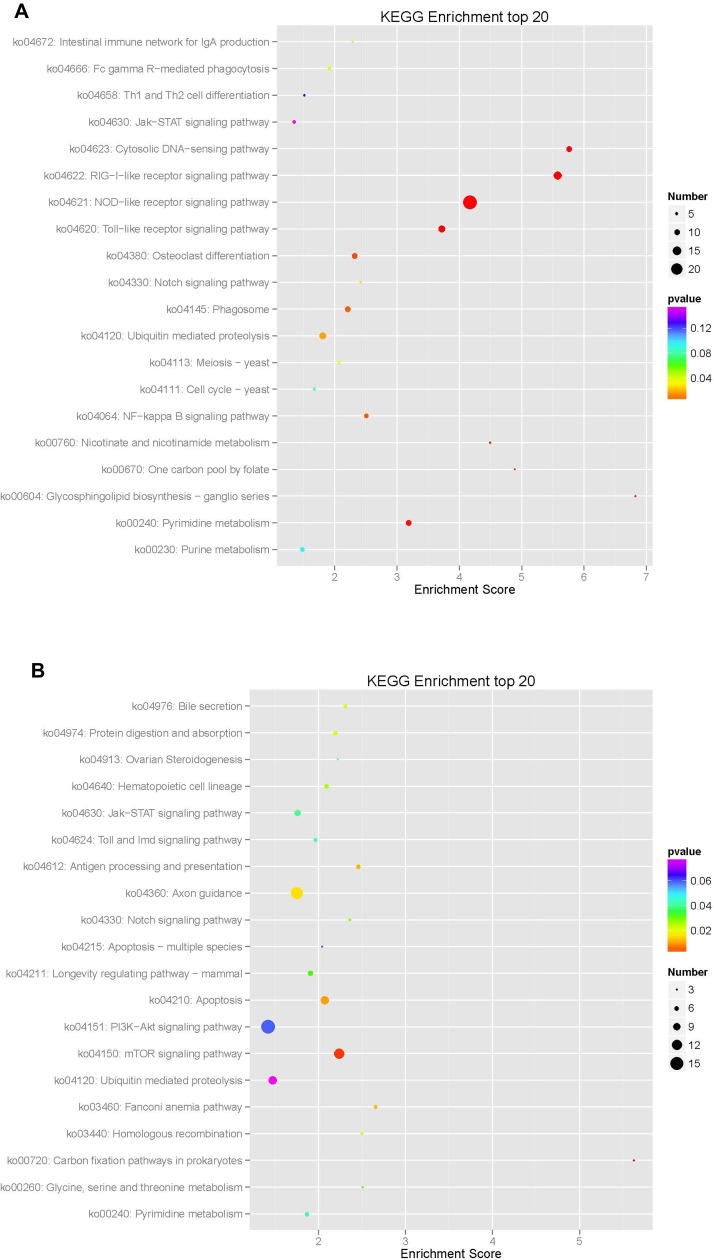
**Enrichment analysis of KEGG pathways in differentially expressed mRNA in macaque mononuclear cells from the juvenile and old age groups.** The enrichment analysis showed that the target gene was mainly enriched in 322 pathways, of which 170 were upregulated and 152 were downregulated, resulting in a top-enriched KEGG pathway map (**A**), downregulated KEGG pathways; and (**B**), upregulated KEGG pathways.

KEGG analysis of the mRNA target genes mainly showed enrichment in 322 pathways, of which 170 were upregulated and 152 were downregulated, showing a top-enriched KEGG pathway map (Figure 14, Table 8). The enriched downregulated KEGG pathways mainly included the NOD-like receptor signaling pathway, the RIG-I-like receptor signaling pathway, the cytosolic DNA-sensing pathway, the Toll-like receptor signaling pathway, nucleotide metabolism-related pyrimidine metabolism, glycosphingolipid biosynthesis-ganglio series related to polysaccharide biosynthesis and metabolism, nicotinate and nicotinamide metabolism associated with cofactor and vitamin metabolism, one carbon pool by folate, growth-related osteoclast differentiation, environmental information processing, and the signal transduction-related NF-kappa B signaling pathways. The enriched upregulated KEGG pathways mainly included the carbon fixation pathways in prokaryotes related to energy metabolism, the mTOR signaling pathway related to environmental information processing and signal transduction, cell growth and apoptosis, antigen processing and presentation related to the immune system, hematopoietic cell lineage, the Fanconi anemia pathway, homologous recombination, growth-related axon guidance, digestion-related digestion and absorption, bile secretion associated with genetic information processing, and replication and repair.

**Table 8:** KEGG pathway analysis of differentially expressed aging-related pathways

Expression	Pathway ID	Pathway name	Gene count	Enrichment score	P-value
Down	ko04621	NOD-like receptor signaling pathway	24	4.17	7.2E-10
Down	ko04622	RIG-I-like receptor signaling pathway	14	5.58	3.6E-08
Down	ko04623	Cytosolic DNA-sensing pathway	10	5.76	1.4E-06
Down	ko04620	Toll-like receptor signaling pathway	12	3.72	2.3E-05
Down	ko00240	Pyrimidine metabolism	10	3.19	0.0003
Down	ko00604	Glycosphingolipid biosynthesis-ganglio series	3	6.82	0.0009
Down	ko00760	Nicotinate and nicotinamide metabolism	4	4.49	0.0020
Down	ko00670	One carbon pool by folate	3	4.89	0.0033
Down	ko04380	Osteoclast differentiation	10	2.32	0.0042
Down	ko04064	NF-kappa B signaling pathway	8	2.50	0.0050
Down	ko04145	Phagosome	10	2.21	0.0060
Down	ko04120	Ubiquitin mediated proteolysis	12	1.81	0.0162
Down	ko04330	Notch signaling pathway	4	2.42	0.0255
Down	ko04113	Meiosis - yeast	5	2.07	0.0350
Down	ko04666	Fc gamma R-mediated phagocytosis	6	1.91	0.0387
Down	ko04672	Intestinal immune network for IgA production	3	2.29	0.0423
Down	ko04111	Cell cycle - yeast	5	1.67	0.0798
Down	ko00230	Purine metabolism	8	1.48	0.0937
Down	ko04658	Th1 and Th2 cell differentiation	4	1.5096	0.1273
Down	ko04630	Jak-STAT signaling pathway	6	1.35	0.1585
Up	ko00720	Carbon fixation pathways in prokaryotes	3	5.63	0.0020
Up	ko04150	mTOR signaling pathway	12	2.24	0.0030
Up	ko04210	Apoptosis	10	2.07	0.0096
Up	ko04612	Antigen processing and presentation	6	2.46	0.0116
Up	ko03460	Fanconi anemia pathway	5	2.65	0.0118
Up	ko04360	Axon guidance	14	1.75	0.0150
Up	ko04974	Protein digestion and absorption	6	2.19	0.0204
Up	ko04976	Bile secretion	5	2.31	0.0220
Up	ko03440	Homologous recombination	4	2.50	0.0224
Up	ko04640	Hematopoietic cell lineage	6	2.09	0.0255
Up	ko04330	Notch signaling pathway	4	2.36	0.0278
Up	ko04211	Longevity regulating pathway - mammal	7	1.91	0.0318
Up	ko00260	Glycine, serine and threonine metabolism	3	2.51	0.0321
Up	ko04630	Jak-STAT signaling pathway	8	1.76	0.0398
Up	ko00240	Pyrimidine metabolism	6	1.87	0.0430
Up	ko04624	Toll and Imd signaling pathway	5	1.96	0.0432
Up	ko04913	Ovarian Steroidogenesis	3	2.22	0.0467
Up	ko04151	PI3K-Akt signaling pathway	16	1.42	0.0599
Up	ko04215	Apoptosis - multiple species	3	2.04	0.0600
Up	ko04120	Ubiquitin mediated proteolysis	10	1.47	0.0796

## DISCUSSION

### Impact of aging on BMSCs

The life expectancy of macaques is approximately 25-30 years and may be longer under artificial breeding conditions. Female macaques are approximately 3 years old and males are 4 years old when they begin to mature. Therefore, macaques are considered young when they are within 3 years of age and considered of old age when they exceed 13 years of age; these guidelines were applied for macaque grouping.

Senescence-associated SA-β-gal is a hydrolase that catalyzes the hydrolysis of β-galactoside to monosaccharides in senescent cells. This hydrolase is therefore considered a biomarker of senescent cells, as is p16^INK4A^ [20, 21]. In this study, SA-β-gal staining was used to quantify the number of senescent cells; and the conditions suitable for inducing cell senescence were evaluated. The aging macaque BMSC model was constructed by treating BMSC with 200 μmol/L t-BHP for 6 h. The overall deterioration of cell stemness and the decrease in MSC activity leading to insufficient functional cell renewal in tissues and organs may be an important mechanism of aging.

BMSCs can secrete a variety of biologically active molecules, including cytokines, chemokines and growth factors, which can promote the repair and regeneration of damaged tissue and organs and maintain many physiological functions, such as homeostasis. In this study, 293T cells were cocultured with BMSCs for 5 d, and the expression of telomeres and the telomerase core protein component TCAB1 was detected. The expression of telomeres and TCAB1 mRNA was significantly increased after coculture of 293T cells with BMSCs. After coculture with BMSCs, it was possible to reverse telomere shortening and improve telomerase activity due to the secretion of various biologically active substances, thereby improving the aging state of the cells.

### Evaluation of the aging macaque BMSC model

In this study, expression of the senescence-associated gene p21 in BMSCs was detected by RT-PCR. The aged group and the induced senescence group had significantly increased levels of p21 compared with the young group. Western blot analysis showed that both p21 and p53 were highly expressed in the aged group and the induced aging group. The results indicated that both groups of cells had entered the aging state.

Sirtuin (SIRT) is a family of proteins homologous to silent information regulator 2 (Sir2), which are also known as antiaging enzymes. The positive role of SIRT1 in combating aging in MSCs has been confirmed. SIRT1 protects cells from aging-related DNA damage, induces the expression of TERT, and enhances telomerase activity [22, 23]. There is a close correlation among SIRT1, telomere length and human life [24]. SIRT1 plays an important role in maintaining the young state of MSCs [25].

SIRT6 is another member of the Sirtuin family with antiaging effects. In this study, SIRT6 was detected by RT-PCR and Western blotting. The expression of SIRT6 in the aged and the induced senescence BMSC groups was significantly lower than that in the juvenile cell group. BMSCs in the aged cell group and the induced senescence group became aged, and the ability of cells to become activated decreased, while accelerated senescence was observed.

TERT is an important substance for stabilizing the length and structure of telomeres. In this study, the expression of TERT and TCAB1 in the aged cell group was significantly lower than that in the juvenile cell group, as observed by RT-PCR analysis of TERT and TCAB1. The telomerase activity of BMSCs was decreased, the telomere length was shortened, and the proliferation potential of cells in vitro was reduced.

BMSCs can secrete a variety of cytokines and play an immunomodulatory role. In this study, the cytokine levels in the cell culture supernatants of each group were detected by ELISA. The cytokine levels in the aged cell group and the induced aging group were significantly lower than those in the juvenile cell group. The results indicated that the aging of cells is accompanied by a decline in paracrine ability, which affects the immune regulation function.

Some authors [26] have found that bone marrow-derived multipotent MSCs are the most frequently investigated cell type for potential regenerative strategies because they are relatively easy to isolate and are able to differentiate into several mesenchymal lineages. These authors concluded that antioxidant supplementation during MSC expansion reduces the DNA damage load and increases the MSC yield.

### Effects of aging on macaques

Increased risk of bacterial infection and chronic inflammation often accompany aging. The proportion of white blood cells and lymphocytes in the peripheral blood of macaques in the elderly group was elevated, suggesting that the old macaques may be in a chronic inflammatory state. The bone marrow supernatant of macaques was analyzed using an angiogenic protein-antibody microarray. The levels of HB-EGF and PIGF in aged macaques were significantly increased, suggesting that the risk of tumors in elderly macaques is increased or that tumors may be present in aged macaques.

Some authors [27] have found that the regeneration potential of MSCs diminishes with advanced age, and this diminished potential is associated with changes in cellular functions. These changes represent novel insights into the aging process and could have implications regarding the potential for autologous stem cell therapy in older patients. In our current manuscript, an aging macaque BMSC model was successfully developed. Both the aged cell group and the induced aging group showed aging morphology; in addition, the expression of aging genes was increased, and the expression of aging-related proteins was increased. The expression trends of the aged cell group and the induced aging group were largely similar. Transcriptome sequencing of macaque mononuclear cells showed that senescence in macaques was related to multiple biological processes, multiple cellular components and multiple molecular functions. Therefore, the novelty of the current manuscript is straightforward.

### Macaque mononuclear cell transcriptome sequencing analysis

Regarding the genomic sequence, the similarity between macaque and human is approximately 93%, and the miRNAs are relatively conserved during evolution; these miRNAs can inhibit gene expression and can have biological significance for tumorigenesis, development, metabolism and viral diseases [28].

Peripheral blood mononuclear cells were sequenced to analyze the differentially expressed mRNA. Through GO enrichment analyses, it was found that the GO terms of the functions of downregulated genes mainly included regulation of the IFN-γ response, defense against virus, the type I interferon signaling pathway, negative regulation of viral genome replication, the innate immune response IFN-γ mediated signaling pathway, IFN-α response, IFN-β response, and negative regulation of type I IFN products. The gene functions involved in the downregulated GO terms are primarily related to the immune system. The immune system is an important defense mechanism of the body against the stress of pathogens. Age-related immune system dysfunction is also called immune aging, which is characterized by increased susceptibility to infectious diseases, risk of developing autoimmune diseases, and risk of tumor occurrence [29]. In general, the downregulated GO terms are closely related to the immune system. Immunosuppression leads to decreased secretion of related cytokines and weakened immune function. The data suggest that the ability of the old macaques to defend against viruses is reduced.

The gene functions involved in the upregulated GO terms mainly included the transport of mevalonate, the behavioral response to nutrition, the spectrin-associated cytoskeleton, the binding of organic cyclic compounds, and the differentiation of erythrocytes. The relationship between these gene functions and aging is still unclear and warrants further investigation. Overall, the downregulation of immune function-related pathways suggests a decrease in immune function in aged macaques, similar to the results of the GO enrichment analysis.

The upregulated KEGG pathways involved the carbon fixation pathways in prokaryotes, the mTOR signaling pathways, and the cell apoptosis pathways. In this study, sequencing analysis showed that the expression of the mTOR signaling pathway was upregulated, suggesting an increased risk of tumors in elderly macaques. Cell apoptosis involves the removal of damaged or redundant cells by activating cysteine-containing aspartate-specific cysteine proteases, and the results suggest that there is an increase in apoptosis in aged macaques.

## MATERIALS AND METHODS

### Detection of the proliferation, senescence, phenotype and differentiation ability of macaque BMSCs

Three macaques with an average age of 2.7 years (2.7 ± 0.58) and three macaques with an average age of 20.7 years (20.7 ± 0.58) were selected for the young and old groups. Bone marrow was collected using the aseptic technique. BMSCs were isolated and cultured by adherent culture screening. The structure and morphological changes were observed under the microscope. The CCK-8 method was used after 7 d of dynamic monitoring to study the old and young macaque P3 BMSCs. The β-galactosidase reagent detected the proportion of senescent cells. Flow cytometry was used to detect the cell surface antigens CD29, CD45, CD73, CD90, CD105, CD184 and HLA-DR. BMSCs were induced for differentiation into bone, fat and chondrocytes. Experimental protocols were approved by the Experimental Animal Ethics Committee of Kunming General Hospital of The People’s Liberation Army.

### Construction and evaluation of the aging macaque BMSC model

Young cynomolgus BMSCs were treated with t-BHP at a concentration of 100-600 μmol/L for 6 h. The senescence ratio of the cells was measured using the β-galactosidase reagent. ELISA was used to detect the levels of IL-6, IL-11 and GM-CSF in young, aged and induced aged BMSC culture supernatants. RT-PCR was used to detect the mRNA expression levels of TERT, TCAB1, P21, SIRT1 and SIRT6 in BMSCs. The expression levels of the aging-associated proteins TCAB1, P21, P53 and SIRT6 in BMSCs were detected by Western blotting.

### Anti-293T cell aging effect of young macaque BMSCs

For this experiment, 293T cells were treated with Tert-butyl hydroperoxide (t-BHP) at a concentration of 0-500 μmol/L for 2 h, and the senescence ratio of the cells was measured using the β-galactosidase reagent. The senescent 293T cells were cultured in the Transwell coculture system together with young macaque BMSCs. The relative expression of the telomere- and telomerase-related gene TBAB1 was detected by RT-PCR. The expression changes of the TCAB1 protein were detected by Western blotting.

### Detection of cytokines in macaque bone marrow tissue and sequencing of the mononuclear cell transcriptome

A protein-antibody microarray was used to detect the levels of angiogenesis-related factors in the supernatant of bone marrow from macaques in the aging and young groups. The mRNA of mononuclear cells in the peripheral blood from macaques in the old and young groups was sequenced by second-generation gene sequencing technology. The sequencing data were analyzed by bioinformatics methods to reveal the differences in transcriptome levels in the young and old groups.

### Statistical analysis

All statistical analyses were performed using SPSS 21.0 statistical software. The measurement results were expressed as the mean ± standard deviation ($\bar{X}\pm \text{s}$). The paired t-test was used for pairwise comparison between groups. Three-group comparisons and the above results were analyzed by one-way analysis of variance (one-way ANOVA). Analysis of the differential expression of transcripts was performed using the negative binomial distribution test for the differential gene significance test of read values. GO analysis and KEGG pathway analysis were performed using the hypergeometric distribution test for significant differential gene enrichment, and P < 0.05 was considered statistically significant.

## CONCLUSIONS

1. The aged cell group is more prone to morphological changes associated with aging when cultured in vitro, and the proliferative ability of the cells in this group is decreased. The number of aged cells in this group is increased, while the multidirectional differentiation ability and the paracrine ability are decreased. MSC stemness is comprehensively decreased, resulting in insufficient functional cell turnover in organs, which may be an important mechanism for aging in the body. Cocultures of aging 293T cells with young macaque BMSCs showed that BMSCs have antiaging effects.

2. An aging macaque BMSC model was successfully developed. Both the aged cell group and the induced aging group showed aging morphology; the expression of aging genes was increased, and the expression of aging-related proteins was increased. The expression trends of the aged cell group and the induced aging group were largely similar.

3. The results of the protein chip experiments suggest that the risk of tumors in old macaques is increased. The peripheral blood cell analysis suggests that the old macaques may be in a chronic inflammatory state and that BMSCs may interact with the microenvironment in vivo.

4. Transcriptome sequencing of macaque mononuclear cells showed that senescence in macaques is related to multiple biological processes, multiple cellular components and multiple molecular functions. The GO terms and KEGG pathways involved in mRNA target gene alteration suggested that the immune functions of old macaques were decreased, the tumor risk was increased, apoptosis was increased, and the involved genes may be related to the aging of macaques. Further research is needed to confirm these results.

## SUPPLEMENTARY MATERIALS

Supplementary Tables
