# Supplementary material for: Relationship between senescence in macaques and bone marrow mesenchymal stem cells and the molecular mechanism
Source: Aging (Albany NY). 2019 Jan 23;11(2):590–614. doi: 10.18632/aging.101762 (PMC6366955; doi:10.18632/aging.101762)
Supplement: Supplementary Tables [file aging-11-101762-s001.pdf]

## SUPPLEMENTARY MATERIAL

**Supplementary Table 1. Primer sequences for telomeres and a control.**

| Gene name | Primer sequence (5'-3') | Amplicon length (bp) |
|---|---|---|
| Telomere | Forward: CGGTTTGTTTGGGTTTGGGTTTGGGTTTGGGTTTGGGTT | >76 |
| | Reverse: GGCTTGCCTTACCCTTACCCTTACCCTTACCCTTACCCT | |
| 36B4 | Forward: CAGCAAGTGGGAAGGTGTAATCC | 75 |
| | Reverse: CCCATTCTATCATCAACGGGTACAA | |

The primer sequence of TCAB1 in Table 2 is shown in Supplementary Material.

**Supplementary Table 2. Primer sequences of tested genes.**

| Gene name | Primer sequence (5'-3') | Amplicon length (bp) |
|---|---|---|
| TERT | Forward: GGAGCAAGTTGCAAAGCATTG | 182 |
| | Reverse: TCCCACGACGTAGTCCATGTT | |
| TCAB1 | Forward: CATATCTGGGACGCATTCACT | 129 |
| | Reverse: GTTGAAGCCACAGAAGAGCTG | |
| P21 | Forward: GAAGCAACAGAGACGGGAAC | 106 |
| | Reverse: CTCATCGCCAACAAGGTAGC | |
| SIRT1 | Forward: TGTGTCATAGGTTAGGTGGTGAA | 130 |
| | Reverse: AGAGGTGTGGGTGGCAAGT | |
| SIRT6 | Forward: CCACCAAGCACGACCGCCAT | 128 |
| | Reverse: CGCCCTCTCCAGCACACGG | |
| GAPDH | Forward: GTCATCAATGGAAATCCCATCA | 98 |
| | Reverse: CCAGTGGACTCCACGACGTAC | |

Primary reagents

**Supplementary Table 3. Manufacturer and model/item No. of reagents.**

| Name | Manufacturer | Model/Item No. |
|---|---|---|
| NC film | US Millipore company | HATF00010 |
| SDS-PAGE gel preparation kit | Shanghai Biyuntian Company | P0012A |
| BCA protein quantification kit | Shanghai Biyuntian Company | P0006 |
| Prestained protein marker | Shanghai Biyuntian Company | P0066 |
| ECL chemiluminescence Kit | Shanghai Biyuntian Company | P0018A |
| Cellular senescence kit β-galactosidase staining kit | Shanghai Biyuntian Company | C0602 |
| WRAP53 polyclonal antibody | American Proteintech | 14761-1-AP |
| SIRT6 polyclonal antibody | American Proteintech | 13572-1-AP |
| P53 monoclonal antibody | US CST Corporation | 2527 |
| P21 monoclonal antibody | Abcam, UK | Ab109199 |
| GAPDH polyclonal antibody | Shanghai Biyuntian Company | AG019 |
| Goat anti-rabbit IgG secondary antibody | Beijing Yiqiao Shenzhou company | SSA004 |
| 0.25% trypsin | American HyClone | SH30042.01 |
| Streptomycin | American HyClone | SV30010 |
| Fetal bovine serum | Israel BI | 04-001-1A |
| DME/F12 medium | American Sigma company | D6421 |
| DMSO | American Sigma company | D4540 |
| TRIzol | US Invitrogen | 15596-018 |
| GoScript reverse transcription system | Promega, USA | A5001 |
| qPCR Master Mix | Promega, USA | A6001 |
| primer | Shanghai Shenggong Company | — |
| Rhesus monkey bone marrow Mesenchymal stem cell culture medium | Guangzhou Cyagen Corporation | MKRMA-90011 |
| Osteogenic differentiation medium | Guangzhou Cyagen Corporation | MKRMA-90021 |
| Adipogenic differentiation medium | Guangzhou Cyagen Corporation | MKRMA-90031 |
| Chondrogenic differentiation medium | Guangzhou Cyagen Corporation | MKRMA-90041 |
| Pentobarbital sodium | Sigma | 57-33-0 |
| CCK-8 | Shanghai DOJINDO Company | CK04-3000T |
| Mouse anti-human CD45 | US BD company | 555555 |
| Mouse anti-human CD73 | US BD company | 550257 |
| Mouse anti-human HLA-ABC | US BD company | 555482 |
| Mouse anti-human CD90 | US eBioscience | 11-0299-42 |
| Mouse anti-human CD105 | US eBioscience | 11-0909-42 |
| Mouse anti-human HLA-DR | US eBioscience | 11-1057-42 |
| IL-11 ELISA kit | Hangzhou Lianke Biological Company | EK1112 |
| GM-CSF ELISA kit | US Proteintech | KE00003 |
| IL-6 ELISA kit | US eBioscience | BMS641/2 |
| Angiogenic antibody arrays | Abcam, UK | Ab197419 |
